# Facial Hemiplegia Treated with Botulinum Toxin: A Case Report

**DOI:** 10.3390/diseases10040067

**Published:** 2022-09-22

**Authors:** Demétrio Ajuz, Mauro D. Oliveira, Juliana Campos Hasse Fernandes, Gustavo Vicentis de Oliveira Fernandes

**Affiliations:** 1 Implant Dentistry, Rio de Janeiro 22031-071, Brazil; 2 Brazilian Academy of Dentistry (AcBO), Rio de Janeiro 22031-071, Brazil; 3 Department of Physiology, Ribeirão Preto Medical School, University of São Paulo, Ribeirão Preto 21710-232, Brazil; 4 Private Office, Ann Arbor, MI 48109, USA; 5 Periodontics and Oral Medicine Department, School of Dentistry, University of Michigan, Ann Arbor, MI 48109, USA

**Keywords:** facial hemiplegia, botulinum toxin, muscles, esthetic, case report

## Abstract

Facial hemiplegia happens when the seventh cranial nerve is inflamed, causing a dysfunction of the facial nerve in specific regions. This case report brings a complex case of facial hemiplegia, a non-temporary lesion caused by a traumatic accident, which had a more conservative approach, treating the patient with botulinum toxin. After explanation of treatment outcomes, the patient favored treatment on a unilateral side with botulinum toxin applied locally to the muscles. It was applied on her left side, in order to change the muscles tonus and improve the esthetic. The patient adhered to immediate and short-term instructions following the procedure, including movement limitation and skin exposure avoidance. At 2 weeks, the patient returned to follow-up, and the result was checked. After around 6-month follow-up, the patient was reassessed, and a new application was done. The patient tried to contract the procerus and corrugator muscles which were treated, and periorbicular region that was corrected. After contracting the frontal muscle, a satisfactory result was also seen in the frontal area. While limited to a single case presentation, botulinum toxin may be an effective short-term tool for treatment of facial hemiplegia to establish an effective esthetic result.

## 1. Introduction

**Figure 1:**
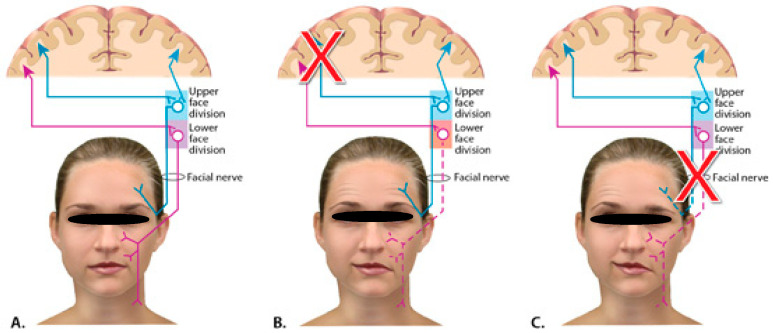
Illustrative nerve pathways from the face to the brain. (**A**). Normal condition. (**B**). Problem is affecting the lower face division. (**C**). Full side affected.

The facial nerves control all the muscles in the face, and they emerge from the middle of the brainstem, carrying motor fibers (from the motor cortex of both cerebral hemispheres) to the facial expression muscles. From their origin, they send fibers to supply the muscles in the upper face, to control eye closure and forehead movement, and in the lower face (mouth). In the brainstem, these fibers cross over to the opposite facial nerve. The upper face fibers take a slightly different path. Half of the fibers travel over to the contralateral facial nerve, and another half remain on the same side, contributing to the ipsilateral facial nerve. Thereby, the eyes and forehead receive innervation from both hemispheres, while the lower face only receives innervation from the contralateral hemisphere (Figure 1) [1].

Facial hemiplegia happens when the seventh cranial nerve is inflamed, causing a dysfunction of the facial nerve (responsible for controlling facial muscles) and affecting facial motor expressions. The weakness in the facial muscles makes one side of the face droop. Bell’s palsy and ischemic stroke are the two most common causes of facial paralysis [2]. Because acute stroke is a time-critical illness, the distinction between stroke and Bell’s palsy must be made quickly to avoid unnecessary delays in treatment.

All those innervations aforementioned are critical when assessing facial weakness. Lesions that damage the motor cortex will result in contralateral facial weakness of only the lower face, preserving the muscles of the upper face on both sides, as shown in Figure 1. Even through that event, patients will be able to close their eyes tightly and wrinkle their forehead symmetrically (central facial weakness), because the damage was caused to the cerebral cortex (part of the central nervous system) [3].

On the other hand, when the lesion damages the facial nerve in the brainstem or after it exits the brainstem, it is referred to as a “peripheral lesion”, resulting in an ipsilateral facial weakness (involving both the upper and lower face). Patients will be unable to wrinkle their forehead, tightly close their eyes, or smile on the affected side [4].

The surgery to correct facial paralysis looks at the improvement of the function and appearance of the face. The professional must consider observing the cause of the paralysis and the areas affected, the person’s general physical and emotional health, the type of procedure(s) most appropriate for the case, and the expected results and possible prognosis, carefully taking into account the patient’s age, medical history, residual hearing, a segment of nerve-injured, and the patient’s expectations and risk tolerance [5].

Regardless of the cause, there is significant complexity in managing facial paralysis, frequently involving a multidisciplinary intervention. Current management consists of a combination of pharmacologic and physical therapy for facial neuromuscular retraining and surgical intervention (dynamic and static techniques) for facial reanimation. Basically, any surgical intervention involves facial nerve decompression, such as in Bell’s palsy or Ramsay Hunt syndrome, or then primary facial nerve repair/grafting in cases of resection or transection of the facial nerve [6].

Then, this case report brings a complex case of facial hemiplegia, a non-temporary lesion caused by a traumatic accident; treatment with an alternate approach using botulinum toxin led to an optimal outcome compared to the previously reported literature.

## 2. Case Report

Patient S.T.A., a female 38 years old, visited the dentist, Dr. D.A., at the Brazilian Academy of Facial Harmonization (Rio de Janeiro, Brazil), in August 2019, pursuing a solution to correct a muscular asymmetry caused by facial hemiplegia in the right side. The patient authorized to use this study’s images and reports (Authorization and Consent Form) and it was followed by the CARE guidelines [7].

**Figure 2:**
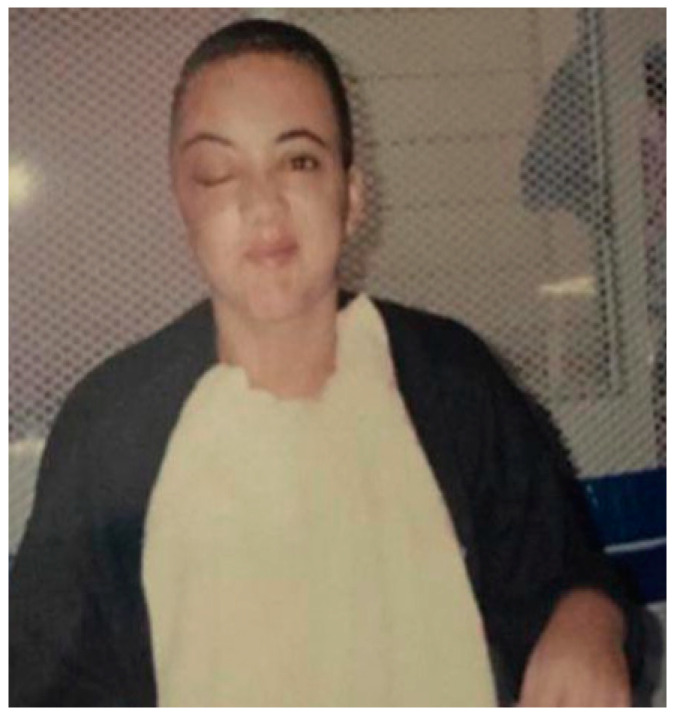
Patient after procedures showing facial hemiplegia on the right side.

The patient was reportedly involved in a motor vehicle accident at an intersection in the city of San Diego (CA, USA) on 13 April 2000, when she was 21 years old. She was taken unconscious to the hospital and was in a coma for 15 days. A computed tomography scan was performed at the hospital (Supplementary Figures S1–S4). Axial cuts and images were made with soft tissue in subdural and bony windows. The findings were: comminuted fractures involving the right orbit, extending to the frontal fossa; right frontal epidural hematoma of moderate size; adjacent pneumocephalus is evident, small subarachnoid hemorrhage on the right side; the brain appears diffusely swollen; the ventricles are compressed; and the basal cisterns slightly effaced. Figure 2 shows the patient at the hospital after procedures.

## 3. Differential Diagnosis

The facial paralysis was not framed within Bell’s palsy (temporary weakness), Ramsay Hunt syndrome, or ischemic stroke. There was a severe nerve lesion due to the trauma suffered, stating the diagnosis as facial hemiplegia.

## 4. Treatment Plan

**Figure 3:**
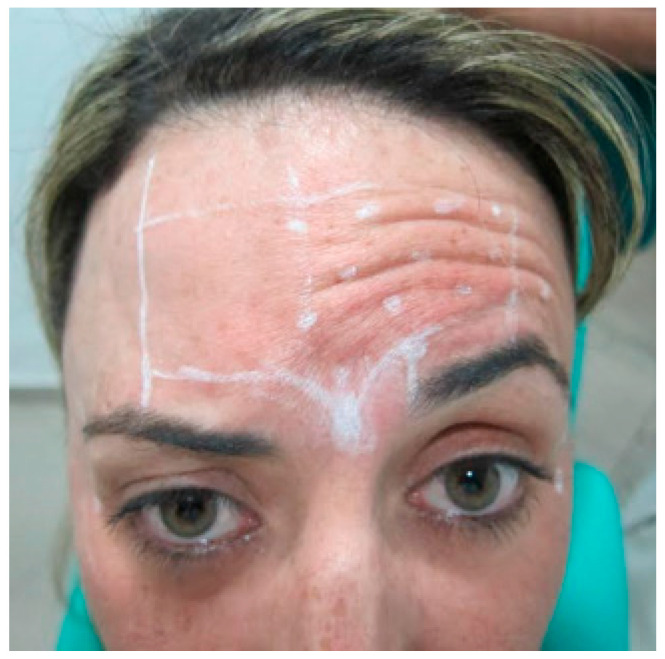
The patient was adequately marked in their limits and safety zones. Individual markings were performed in the active region of the frontal muscle, with 1 to 1.5 cm of distance, and 2U of the toxin was injected (Botulift 100U). A central point was made in the procerus muscle, and 6U was injected. Then, 4U was injected in the corrugator muscle of the eyebrow on the left side.

After explanation of treatment outcomes, the patient favored treatment on a unilateral side with botulinum toxin applied locally to the muscles (Figure 3). All pros and cons were explained to the patient, mainly that this type of treatment typically loses the effectivity after 4–6 months. The patient adhered to immediate and short-term instructions following the procedure, including movement limitation and skin exposure avoidance.

## 5. Treatment

**Figure 4:**
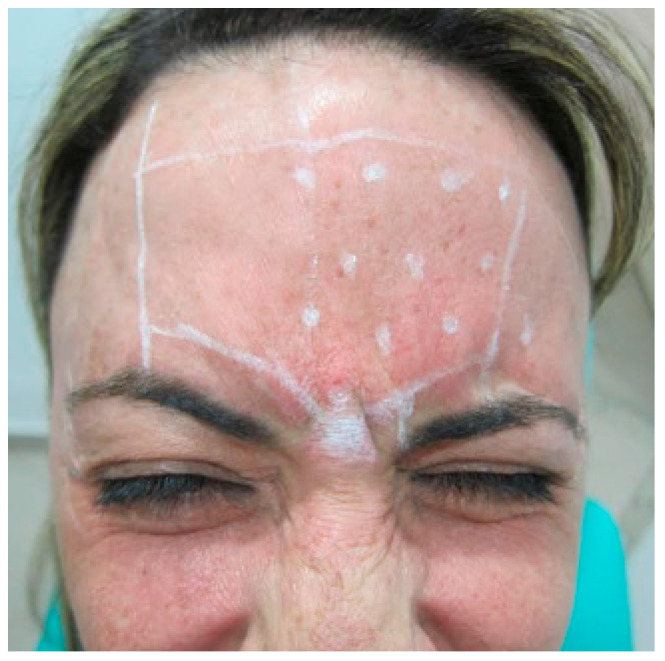
Points delimiting the area of application.

The patient was duly marked in their limits and safety zones. Individual markings were performed in the active regions of the procerus and corrugator muscles of the eyebrow, the latter on the left side. A central point was made in the procerus muscle, 6U was injected, and 4U was injected in the corrugator muscle of the eyebrow on the left side (Figure 4).

**Figure 5:**
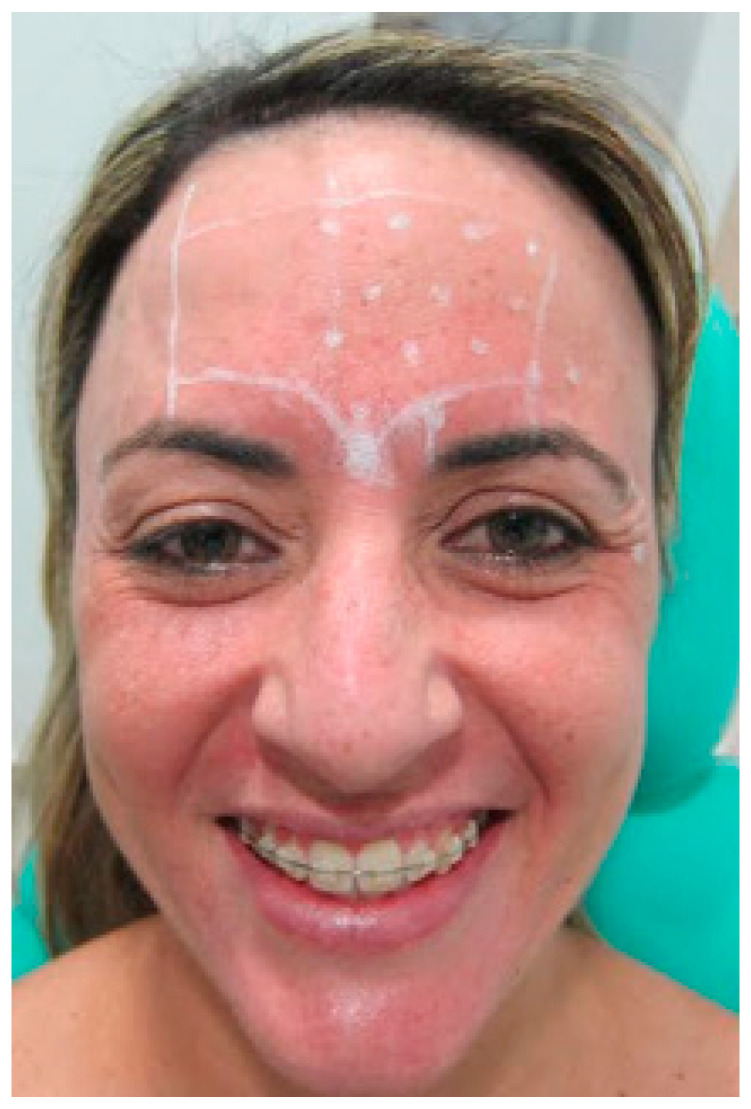
At 2-week follow-up, the patient was noted to have correction of complex facial muscle function and symmetrical appearance of the frontal area with frontal muscle contraction (Figure 6 and Figure 7).

**Figure 6:**
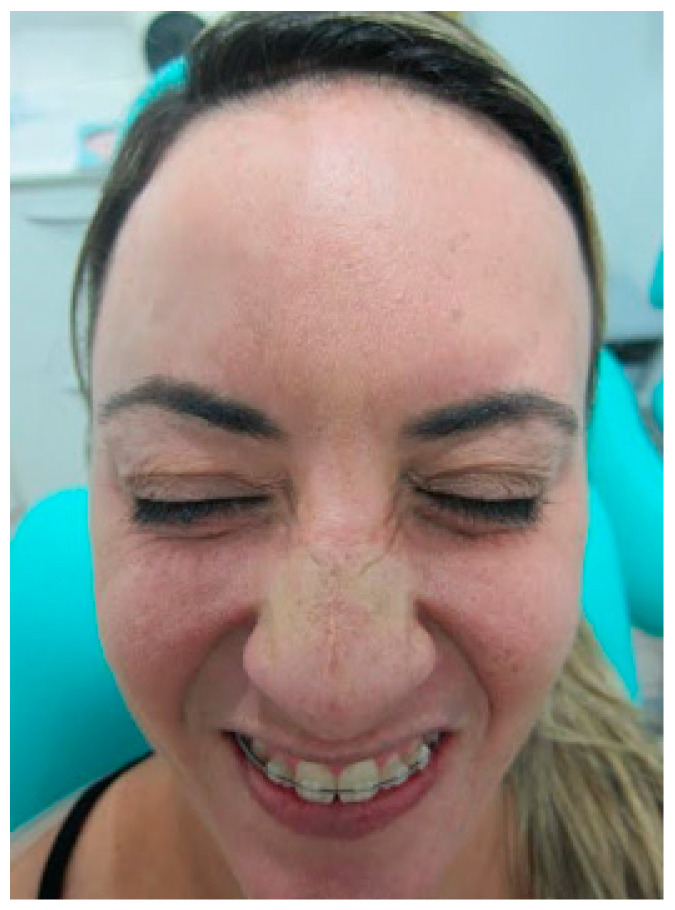
After 14 days, contraction of the procerus and corrugator muscles shows an equilibrium.

**Figure 7:**
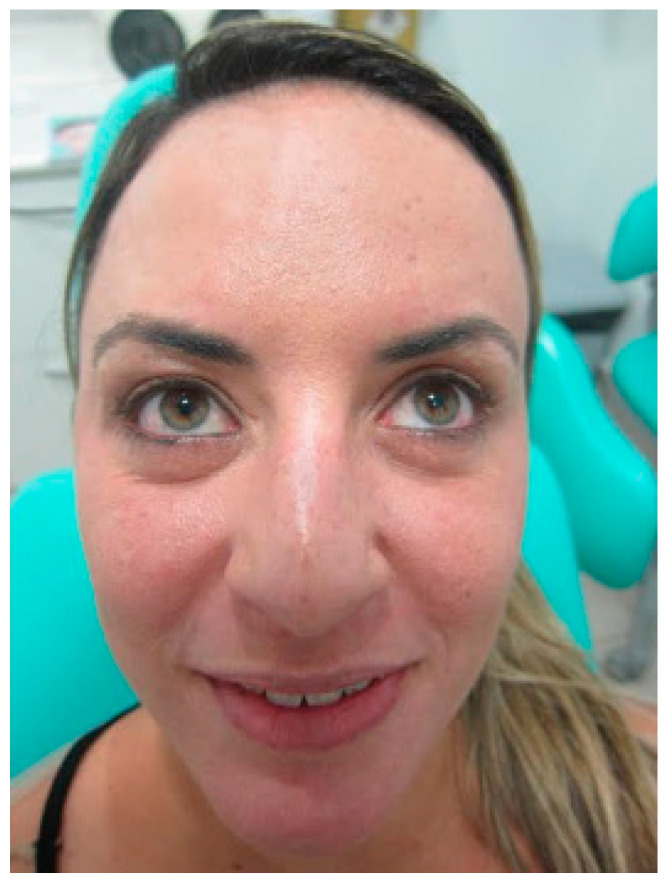
After 14 days, contraction of the frontal muscle was corrected.

It was requested that the patient give a smile, forcing the peri-orbicular region on the left side to decrease and adjust asymmetry. The patient was properly marked in their limits and safety zones. Only one mark was performed in the distal portion in the active region of the orbicularis oculi muscle. Then, 2U of botulinum toxin was applied only on the left side (Figure 5, Figure 6 and Figure 7).

The patient declared to be completely satisfied with the result and treatment. Moreover, she reported that the cost-benefit of the treatment without a traumatic intervention was a better solution, even with the botulinum toxin’s temporary effect.

**Figure 8:**
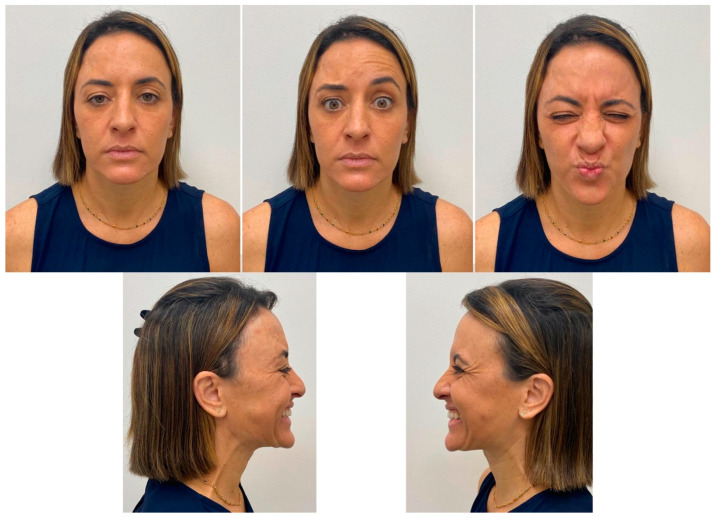
Re-evaluation of the patient after more than 6 months. Observe the difference of the muscle’s activity.

**Figure 9:**
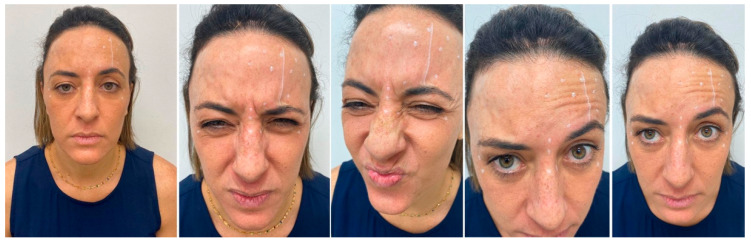
Clinical evaluation for a new application of botulinum toxin.

**Figure 10:**
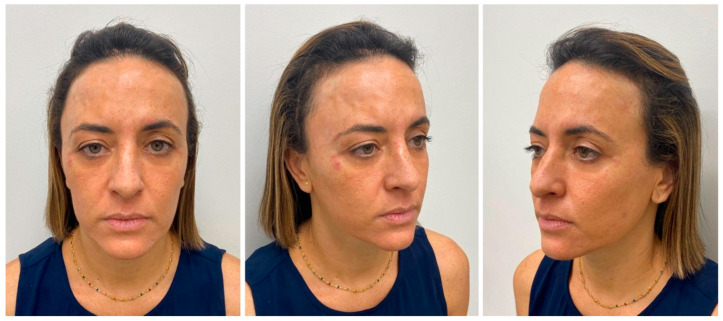
Immediate result after the second application.

After 6 months passed, the patient returned for reassessment (Figure 8). New application of botulinum toxin was done to reduce the muscle’s activity (Figure 9). The result after new application showed better similarity in the muscle’s action, improving the esthetic (Figure 10). At once, the patient reported to be very satisfied with this type of intervention.

## 6. Discussion

To the best of our knowledge, this is the first case report using botulinum toxin to treat a case of facial non-temporary hemiplegia. This approach permitted a temporary solution without traumatic intervention, which can be interpreted by the patients as a first tentative of non-surgical treatment, obtaining an interesting esthetic result.

Traditionally, many surgical procedures can be analyzed. The treatment may involve facial nerve decompression surgery in cases of Bell’s palsy or Ramsay Hunt syndrome (viral-induced facial paralysis) or primary facial nerve repair and grafting in cases of resection or transection of the facial nerve if the paralysis persists for up to 3 weeks. Another approach can be suggested between 3 weeks and 2 years, such as nerve transfers and cross-facial nerve grafting. The approaches may include local and free muscle transfer for problems lasting more than 2 years, considered chronic facial paralysis [6]. Therefore, those approaches still have questionable predictability of success and prognosis.

Another alternative approach was reported by Tong et al., presenting acupuncture at key acupoints combined with rehabilitation therapy to relieve the spasmodic condition in case of hemiplegia. The results were effective and significantly improved the life activity of the patients [8].

Botulinum toxin was assessed in spasticity and motor performance in chronic stroke with spastic hemiplegia. Ten stroke survivors with spastic hemiplegia received 100 units of incobotulinumtoxinA or onabotulinumtoxinA to the biceps brachii muscles. The patients were evaluated after 3–4 weeks, and the result provided evidence that botulinum toxin injections can reduce spasticity and muscle strength [9]. Furthermore, a systematic review analyzing Botulinum toxin in treatment of facial palsy, including just three articles, reported that Botulinum toxin injections are a minimally invasive technique that is helpful in restoring facial symmetry, and its combination with physical therapy may be particularly helpful, therefore concluding that more research is suggested, and the recommendation of botulinum may be a feasible option of facial palsy treatment [10].

In this study, the focus was different, as the contralateral side was treated to obtain esthetic and better performance. The results, even though temporary (suggested effect between 4 and 6 months), were clinically satisfactory and the acceptance and feedback of the patient were high and positive, respectively. However, there are some risks for the incorrect use of botulinum toxin, which must be observed before trying to use, involving majority injection site adverse effects, such as pain, dry skin and subsequent flakiness, injection trauma, infection, scar (rare), allergic symptoms, headache (common), hypoesthesia, paresthesia, dysesthesia (rare), dry mouth sensation, flu-like mild malaise, ecchymosis or bruising (results from injuring a blood vessel), and hematoma (it can last for a longer period; to prevent against abscess, it requires antibiotic therapy). Pressure and ice packs on the injection site should be applied immediately after blood vessel injuries to reduce ecchymosis and bruising. Precautions and suggested recommendations for patients include avoiding medications that inhibit clotting such as vitamin E, aspirin, and nonsteroidal anti-inflammatory drugs (NSAIDs) for a period of 10–14 days prior to treatment to minimize the incidence of bruising [11,12,13].

Moreover, there were limitations in this case report. Even though the patient is aware of the result, the botulinum toxin is a palliative and temporary solution, which may lose its effect after four to six months. The patient must be advised about this question prior to performing the treatment. The patient’s expectation of the result was low because she had had this problem for a long time (more than 19 years). Each case must be individually studied and analyzed because the result cannot be the same, and the approach depends on the type of problem (diagnosis).

## 7. Conclusions

While limited to a single case presentation, botulinum toxin may be an effective short-term tool for treatment of facial hemiplegia to establish an effective esthetic result. Therefore, each case must be carefully studied, and all treatment options must be explained before any type of procedure is performed.

## Supplementary Materials

The following supporting information can be downloaded at: https://www.mdpi.com/article/10.3390/diseases10040067/s1, Figures S1–S4: Report of the computed tomography performed at the moment of the accident (April 2000), pre- and post-op.
