# Supplementary material for: Facial Hemiplegia Treated with Botulinum Toxin: A Case Report
Source: Diseases. 2022 Sep 22;10(4):67. doi: 10.3390/diseases10040067 (PMC9590075; doi:10.3390/diseases10040067)
Supplement: Supplementary file 1 [file diseases-10-00067-s001.zip › diseases-1903343-supplementary.pdf]

Supplemental Material

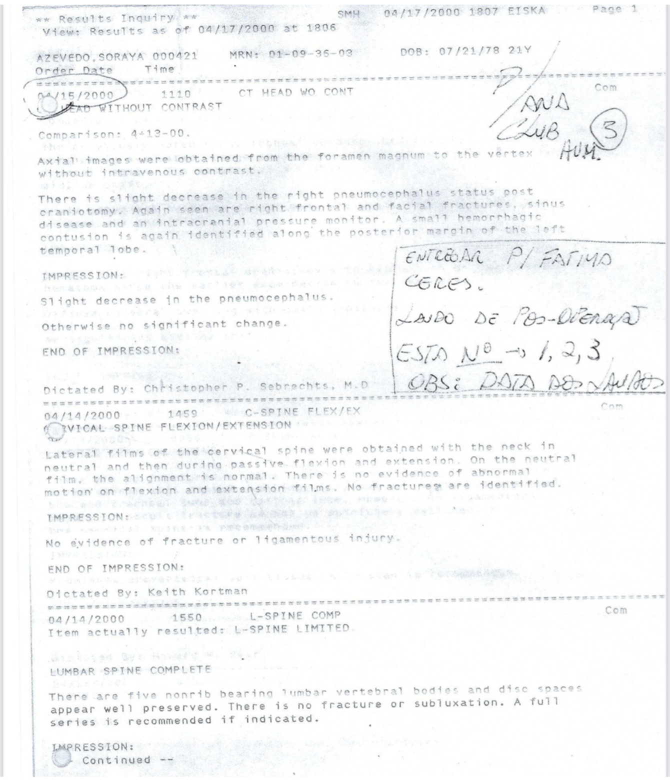
** Results Inquiry ** SMH 04/17/2000 1807 EISKA Page 1
View: Results as of 04/17/2000 at 1806

AZEVEDO,SORAYA 000421 MRN: 01-09-36-03 DOB: 07/21/78 21Y
Order Date Time

04/15/2000 1110 CT HEAD WO CONT Com
HEAD WITHOUT CONTRAST

Comparison: 4-13-00.

Axial images were obtained from the foramen magnum to the vertex without intravenous contrast.

There is slight decrease in the right pneumocephalus status post craniotomy. Again seen are right frontal and facial fractures, sinus disease and an intracranial pressure monitor. A small hemorrhagic contusion is again identified along the posterior margin of the left temporal lobe.

IMPRESSION:

Slight decrease in the pneumocephalus.

Otherwise no significant change.

END OF IMPRESSION:

Dictated By: Christopher P. Sebrechts, M.D

04/14/2000 1459 C-SPINE FLEX/EX Com
CERVICAL SPINE FLEXION/EXTENSION

Lateral films of the cervical spine were obtained with the neck in neutral and then during passive flexion and extension. On the neutral film, the alignment is normal. There is no evidence of abnormal motion on flexion and extension films. No fractures are identified.

IMPRESSION:

No evidence of fracture or ligamentous injury.

END OF IMPRESSION:

Dictated By: Keith Kortman

04/14/2000 1550 L-SPINE COMP Com
Item actually resulted: L-SPINE LIMITED

LUMBAR SPINE COMPLETE

There are five nonrib bearing lumbar vertebral bodies and disc spaces appear well preserved. There is no fracture or subluxation. A full series is recommended if indicated.

IMPRESSION:
-- Continued --

Figure S1. Report of the computed tomography performed at the moment of the accident (April 2000), pre- and post-op. (1).

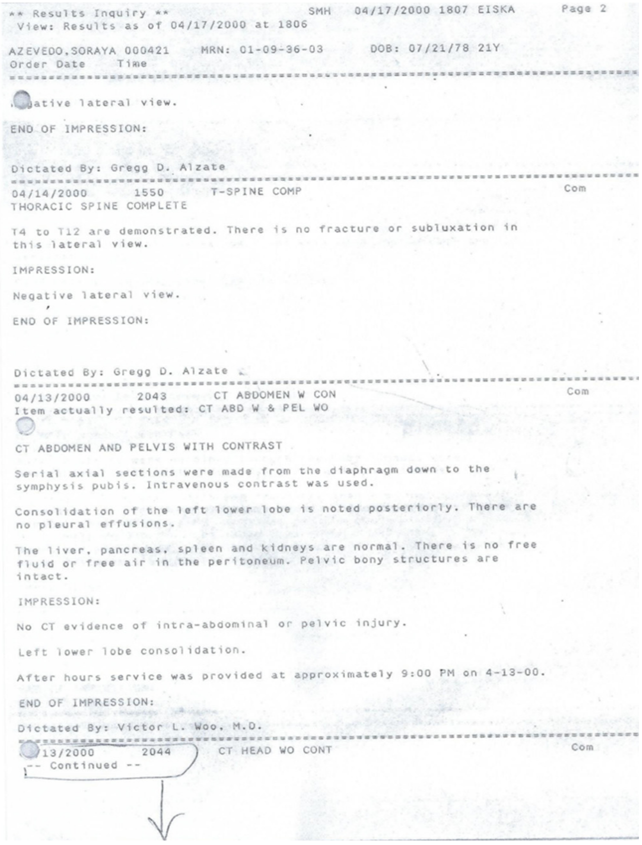
** Results Inquiry ** SMH 04/17/2000 1807 EISKA Page 2
View: Results as of 04/17/2000 at 1806

AZEVEDO,SORAYA 000421 MRN: 01-09-36-03 DOB: 07/21/78 21Y
Order Date Time

[illegible]ative lateral view.

END OF IMPRESSION:

Dictated By: Gregg D. Alzate

04/14/2000 1550 T-SPINE COMP Com
THORACIC SPINE COMPLETE

T4 to T12 are demonstrated. There is no fracture or subluxation in this lateral view.

IMPRESSION:

Negative lateral view.

END OF IMPRESSION:

Dictated By: Gregg D. Alzate

04/13/2000 2043 CT ABDOMEN W CON Com
Item actually resulted: CT ABD W & PEL WO

CT ABDOMEN AND PELVIS WITH CONTRAST

Serial axial sections were made from the diaphragm down to the symphysis pubis. Intravenous contrast was used.

Consolidation of the left lower lobe is noted posteriorly. There are no pleural effusions.

The liver, pancreas, spleen and kidneys are normal. There is no free fluid or free air in the peritoneum. Pelvic bony structures are intact.

IMPRESSION:

No CT evidence of intra-abdominal or pelvic injury.

Left lower lobe consolidation.

After hours service was provided at approximately 9:00 PM on 4-13-00.

END OF IMPRESSION:

Dictated By: Victor L. Woo, M.D.

[illegible]13/2000 2044 CT HEAD WO CONT Com
-- Continued --

Figure S2. Report of the computed tomography performed at the moment of the accident (April 2000), pre- and post-op. (2).

```
** Results Inquiry **                    SMH    04/17/2000 1807 EISKA       Page 3
 View: Results as of 04/17/2000 at 1806

AZEVEDO,SORAYA 000421     MRN: 01-09-36-03        DOB: 07/21/78 21Y
Order Date    Time
================================================================================
CT HEAD WITHOUT CONTRAST                                          post-op
                                                                     (2)
Comparison: Earlier examination of the same day.

The previously noted right frontal epidural hematoma has been
surgically evacuated. Large pneumocephalus is now noted in the right
frontal region. The ventricles are small. There is no significant
midline shift.

Again noted are multiple facial bone and skull fractures. All the
sinuses are opacified.

IMPRESSION:

Status post right frontal craniotomy with evacuation of the epidural
hematoma since the earlier examination on the same day.

Diffuse cerebral swelling with small ventricles.

No significant midline shift.

END OF IMPRESSION:

Dictated By: Victor L. Woo, M.D.
================================================================================
04/13/2000      0958        C-SPINE AP/LAT                                 Com
LIMITED CERVICAL SPINE

There are no fractures or other bony abnormalities. There is some
prevertebral soft tissue swelling. This may be related to placement of
the endotracheal tube and gastric tube. However, no ligamentous
injury or occult fracture cannot be absolutely excluded. A CT scan of
the cervical spine is recommended.

IMPRESSION:

Prominent prevertebral soft tissue. A CT scan is recommended.

END OF IMPRESSION:

Dictated By: Howard M. Bear
================================================================================
04/13/2000      0957        PELVIS LTD AP                                  Com
LIMITED PELVIS

IMPRESSION:

There are no fractures or other bony abnormalities.
   Continued --
```

Figure S3. Report of the computed tomography performed at the moment of the accident (April 2000), pre- and post-op. (3).

```
** Results Inquiry **                    SMH    04/17/2000 1807 EISKA       Page 4
 View: Results as of 04/17/2000 at 1806

AZEVEDO,SORAYA 000421     MRN: 01-09-36-03        DOB: 07/21/78 21Y
Order Date    Time
================================================================================
[illegible] OF IMPRESSION:

Dictated By: Howard M. Bear
================================================================================
04/13/2000      0950        CT C-SPINE WO CON                              Com
CT CERVICAL SPINE WO CONTRAST

Indication: Traumatic neck injury.

Procedure: 3 mm thick axial sections were obtained through the
cervical spine.

Findings: No abnormalities are identified.

IMPRESSION:

Normal.

END OF IMPRESSION:

Dictated By: Keith Kortman
================================================================================
[illegible]/13/2000     0919        CT HEAD WO CONT      pre-op.                Com
CT HEAD WITHOUT CONTRAST
                                                                     (1)
Axial sections were obtained through the head. Images were
photographed with soft tissue, subdural and bone windows.

Findings: There are comminuted fractures involving the right orbit
extending into the frontal fossa. There is a moderate sized right
frontal epidural hematoma. Adjacent pneumocephalus is evident. There
is a small amount of right-sided subarachnoid hemorrhage. The brain
appears diffusely swollen. The ventricles are compressed and the
basal cisterns are mildly effaced.

IMPRESSION:

Right frontal and facial fractures.

Right frontal epidural hematoma and pneumocephalus.

Diffuse cerebral swelling.

END OF IMPRESSION:

Dictated By: Keith Kortman
================================================================================
                                                     -- End of Report --
```

Figure S4. Report of the computed tomography performed at the moment of the accident (April 2000), pre- and post-op. (4).
